# Pyorrhœa Alveolaris—Personal Experience in Treatment

**Published:** 1899-01

**Authors:** W. Geo. Beers

**Affiliations:** MONTREAL.


ARTICLE II.

### PYORRHŒA ALVEOLARIS.—PERSONAL EXPERIENCE IN TREATMENT.

W. GEO. BEERS, L. D. S., D. D. S., MONTREAL.

Read before the Maritime Dental Association, September, 1898.

What we positively know about the pathology of pyorrhœa alveolaris might cover half a sheet of note paper. What we positively do not know would fill folios. Our investigations are like a deep dive into a dark sea, knowing nothing of the bottom, and uncertain of rescue; and while the regulation " twenty minutes " ought to suffice to tell all I know of my own experience in treatment, it would take hours to relate explorations in the realms of pathological speculation, and the many discoveries made of one's own etiological ignorance. We make a step towards knowledge when we discover and confess our ignorance, and perhaps one's frankness in this direction may be a little inspired by the feeling that his friends are in the same plight. Somebody said that while caries is the most prevalent disease in existence, there are more teeth lost by pyorrhæa alveolaris; but we have no statistical proof that this is true, and I am disposed to accept it as one of those hasty and epigrammatic statements, which pass too current entirely because of their catching phraseology. We cannot assume that all teeth that are "lost" are lost legitimately. There are more legitimate deaths than births. It is quite safe to suggest that thousands of teeth are "lost" by ignorance on the part of the patient or malpractice on the part of the dentist. It needs no extensive experience to assert, that the large proportion of "lost" teeth in the establishment of the quack dentist, are like lost souls that have none but themselves to blame for their damnation.

We need no statistical proof to declare that the diseases which attack the soft and adjacent structures of the mouth, especially those intimately connected with the alveoli, are more difficult to treat, and more likely to recur, than those which attack the teeth themselves. The force of resistence (*vis medicatrix naturæ*) aid the soft structures; it is entire absent in the hard, unless in the very exceptional and limited cases of arrested caries. Yet there is a limit to the tolerance which the soft structures exhibit, and when that limit is passed we observe irretrievable recession of gum, alveolar margins, entire destruction of the sockets and pericementum. There are a hundred questions which might be referred to, but however we may speculate on the etiology of this disease, I venture to express my belief, that if we can take care of the gingival line, where the enamel and cementum join, or, in brief, of the gum tissue at the immediate neck of the tooth, the rest of the gum will largely take care of itself. In modern dental practice the abuses of the free margin of the gum are as daring as they are dangerous. It was not so in the olden time. The wisdom which only comes by experience, is entirely absent in the army of young enthusiasts who revel in rubber dam, ligatures, clamp wedges, and who rush into the operating arena with all the devices of the dental engine, with its rapidity of movement and the many abuses with which it is charged. Overhanging fillings, the nidus they make for debris pressing on the interproximate spaces; the numerous mechanical injuries inflicted on the gingival line by clasps, unfinished margins of vulcanite pressing on the interproximate spaces, so as to make deep pockets and ultimate gum recession—these are very ordinary exciting factors in bringing about the first stage of pyorrhœa. The interdependence of the teeth and gums is imperative. The teeth will be retained in the socket, it may be for years, but it is never forever. The gums will tolerate an immense amount of mechanical and surgical abuse anywhere better than at the gingival line. Why is this? Because the soft glandular-like epithalium—though it is not a glandular structure—which lies at the neck of the tooth, is composed of more delicate cells than elsewhere in the gums; its fibres are more intimately connected with the cementum; the cementum is thinest as it reaches the neck of the tooth, and the peridental membrane depends for its tense hold on the roots upon the integrity of the fibres of the pericementum which unite the cementum at the neck.

I believe there are constitutional predisposing causes of pyorrhœa alveolaris, but in searching and speculating in that direction, it is wise to search carefully for causes closer to view. We see mouths not only after or during illness, but in good health, which are human cesspools. The origin of a good deal of pyorrhœa alveolaris is no more obscure than the origin of a good deal of typhoid. Many people will tolerate more positive filth, and more pyogenic baeteria in their mouths than they would on their feet.

The etiology of caries is no longer a speculation; that of pyorrhœa alveolaris is entire so. We know that it is a perversion of normal physiological action; that it is a deviation from the normal standard of health of the parts concerned, constituting a distinct disease that may end in the death and loss of the teeth. But we are not agreed as to whether or not it is infectious, local, constitutional, or both. It is a much named disease, whether it be called Rigg's Pyorrhœa Alveolaris, Suppurative Inflammation, Phagedenic Pericementitis. Is it due to salivary or sanguinary calculus, or both, or fungi, or bacteria? Is it wholly or in part due to traumatic inflammation? Is it a local expression of a systemic condition, and located in the mouth because of a predisposition of the gums, the pericementum and the alveoli? Is it a primary or a secondary lesion? Is it never present, excepting in a depraved state of the nervous system? Is it associated with gout, rheumatism, Bright's disease, locomotor ataxia, uterine troubles, and other constitutional complications; the excessive use of salt, or alcohol, which causes increased secretion of uric acid? Is the calculus uric acid? Is it connected with catarrh of the mucus membrane of the nose or pharynx, rachitis, tuberculosis, scorbutis, scrofula, chronic constipation, exanthematous diseases, malaria, diabetes, tabes dorsalis, dyspepsia, syphilis, anæmia, chlorosis, repeated pregnancy, bad air, bad food, hygienic neglect? Or will some Boston prophet arise, who will tell us that it and all other oral troubles are due to the presence of amalgam, as in the middle ages, earthquakes, tempests and epidemics were ascribed to the devil or the Jews. With all this bewilderment in etiology, how can our treatment be based upon anything better than empiricism? If the etiology is obscure, how can treatment be scientific? We must just console ourselves by feeling our way in the dark, and taking the consequences until some one lets in the light. Something must be done for our patients. The true cause of many a disease is obscure, but physicians do not despair and let the patients die if they can help it. Neither are we to abandon to the forceps, if we can help it, conditions we cannot explain. Not even because we are sure of this much, that extraction always cures. So does the guillotine forever cure migraine.

Before leaving this part of the subject, I would like to ask you to obtain Dr. W. C. Barrett's "Oral Pathology and Practice," just issued, in which the question is carefully considered.

It is a well known fact that there are general diseases successfully treated, the causes of which are as unknown as that of pyorrhœa. It is nothing to boast about that this has and can be done; it upsets our theories and teaching in the recognition of disease and its proper treatment.

I like Dr. Barrett's division of the disease into three conditions: the first entirely local, due to local irritation; the second distinguished by the nodular deposits on the root, and the formation of pockets; the third, due to, or coincident with, a depraved constitutional condition. The first is an advanced condition, of simple gingivitis; slight periostitis confined to the gingival margin, and the edge of the alveolus, demanding gentle brushing, massage with the finger. As a regular mouth wash for this condition I prefer tincture of pyrethrum. The pyrethrum root may be left in the best alcohol for a week. It is, I think, the best astringent we possess. It is well to remember that there is a time in this disease when it may be easily arrested, and that there is a time when it is too late. It is well to remember also, that by over treatment we may actually produce to order acute inflammation from the simple irritation. So far as instrumental treatment is concerned, if there is no salivary calculus, a good deal of wholesome neglect is advisable. There is nothing the matter, in this stage, with either the root or the alveoli.

I repeat everything that has been suggested in the way of treatment where the disease has all the pathognomonic characteristics; but, to be as brief as possible, let me suggest a few aphorisms. Keep the mouth and your hands and whatever you use perfectly aseptic. Whatever drugs you use be sure they are pure. You must needs work a good deal in the dark, but get all the light possible, both in diagnosis and in operating. A drug that is useful may be abused by over-use so as to become a positive irritant. Iodine and aconite in the incipient stage of inflammation will retard the circulation and stimulate lymphatic action, but used to excess, as it commonly is, it is a destructive poison. Alkalies in any form should not be used, either by the patient or the operator, because they precipitate the salts of common tartars. In secondary treatment, and, in fact, all through, be careful not to disturb granulations which you have been trying to produce. Do not operate oftener, as a risk, than three days of each week; some rest is as necessary as some operating.

Before beginning the removal of serumal calculus, be as sure as possible just where it lies. There may not be any. I have seen acid conditions, in pregnancy, etc., when salivary calculus for the time is dissolved and disappears. As to whether or not this occurs where the calculus is serumal. I do not know. Massage the gum with the finger to make it bleed, if it will; sometimes lance; sometimes use a leech. Relieve venous congestion as much as possible before scaling. There is an object in manipulating the gum margins so as not to wound them any more than they are already wounded. I begin my diagnosis for the nodules of calculus by explorers I have made for the purpose, using a diagram of the roots to mark the parts upon which it is deposited. My pocket-hoes and scrapers I have had made so as to adapt them as nearly as possible to the contour of the roots. Square hoes and scrapers are not the best. I have had these made in duplicate of platinum-iridium so as to use them when the sulphuric acid is injected into the pockets. Some authorities object to the use of chemical agents to dissolve the deposits, and favor trichloracetic acid to soften them. I venture to believe what is considered an objection, viz., that they may cause a slight dissolution of the surrounding bone, to be advantageous, within proper limitations. Pure sulphuric acid is less destructive than dilute. Dip a wooden point in pure concentrated sulphuric acid; fill the pockets. You can see the advantage here of having the concave platinum-iridium instument. Aromatic sulphuric acid dissolves dead, not live bone, but I believe we overlook the condition of the contiguous alveolar wall, which I always scrape more or less. The dental engine, to my mind, is risky, unless to curette the alveolor border where it is necrosed. If it is necrosed, the surgeon's maxim should be remembered—"cut beyond the dead line." I have had some opportunities to examine the alveolus and teeth *post mortem*, and hope to report later. If anyone else has had a like opportunity, I hope they will report before I do. I think the condition of the porous alveolar wall in pyorrhœa alveolaris has been somewhat overlooked. The nodules on the root may be removed, but their effect on the pericementum alveolus is serious.

For many years the use of sulphate of copper, packed into the pockets twice a week, and allowed to remain for ten minutes each time, has been in favor in England. I swear by it. It contracts spongy gums and makes scaling easier. Its action involves less loss of tissue, while its curative powers are very marked. It does not blacken the teeth like nitrate of silver, or act on them like acids; it does not spread over more surface than desired, like chloride of zinc, caustic, potash, and other such remedies. It causes little pain. Other caustics check granulation. Use bicarbonate of soda as a rinse, and pack it on top of the sulphate before rinsing. Protect the lips with an old napkin.

I have purposely avoided saying a great deal that might, and perhaps ought to be said, about peroxide of hydrogen, etc., and as I wish to leave room for somebody else to say something, I will say no more.—*Dominion Dental Journal.*

